# Genetic Diversity and the Spatio-Temporal Analyses of Hantaviruses in Shandong Province, China

**DOI:** 10.3389/fmicb.2018.02771

**Published:** 2018-11-20

**Authors:** Shu-Qing Zuo, Xiu-Jun Li, Zhi-Qiang Wang, Jia-Fu Jiang, Li-Qun Fang, Wen-Hui Zhang, Jiu-Song Zhang, Qiu-Min Zhao, Wu-Chun Cao

**Affiliations:** ^1^State Key Laboratory of Pathogen and Biosecurity, Beijing Institute of Microbiology and Epidemiology, Beijing, China; ^2^Department of Biostatistics, School of Public Health, Shandong University, Jinan, China; ^3^Shandong Center for Disease Control and Prevention, Jinan, China

**Keywords:** hantavirus, phylogenetic analysis, phylogeny-trait association, phylodynamic, selection pressure

## Abstract

Hemorrhagic fever with renal syndrome (HFRS) is a serious public health problem in Shandong Province, China. We conducted an epizootiologic investigation and phylogeographic and phylodynamic analyses to infer the phylogenetic relationships of hantaviruses in space and time, and gain further insights into their evolutionary dynamics in Shandong Province. Our data indicated that the Seoul virus (SEOV) is distributed throughout Shandong, whereas Hantaan virus (HTNV) co-circulates with SEOV in the eastern and southern areas of Shandong. Their distribution showed strong geographic clustering. In addition, our analyses indicated multiple evolutionary paths, long-distance transmission, and demographic expansion events for SEOV in some areas. Selection pressure analyses revealed that negative selection on hantaviruses acted as the principal evolutionary force, whereas a little evidence of positive selection exists. We found that several positively selected sites were located within major functional regions and indicated the importance of these residues for adaptive evolution of hantaviruses.

## Introduction

Hantaviruses (genus *Orthohantavirus*, family *Hantaviridae*, Order *Bunyavirales*) are among the most widely distributed groups of zoonotic viruses and have been detected in Asia, Europe, the Americas, and Africa (Mir, 2010). They are the causative agents of hemorrhagic fever with renal syndrome (HFRS) in Eurasia, and hantavirus cardiopulmonary syndrome (HCPS) in the Americas. Hantaviruses are negative-stranded RNA viruses with a tripartite genome, consisting of large (L), medium (M), and small (S) segments, encoding RNA-dependent-RNA polymerase (L), membrane glycoproteins (Gn and Gc), and nucleocapsid protein (N), respectively. Each hantavirus species is primarily associated with one or a few closely related rodent, insectivore, or bat species in nature (Zeier et al., 2005; Jonsson et al., 2010). Humans usually acquire hantavirus infection by contact or inhalation of aerosols and secretions from infected rodent hosts.

HFRS is endemic in China and remains a serious public health problem with about 10,000 human cases annually in recent years (Zhang et al., 2010, 2014; Jiang et al., 2016), even though many measures such as environmental management, host surveillance, and HFRS vaccine implementation have been carried out to control the disease. In China, the Hantaan virus (HTNV) and Seoul virus (SEOV), mainly carried by *Apodemus agrarius* and *Rattus norvegicus*, respectively, are the predominant causative agents of HFRS. The common symptoms of HFRS include headache, myalgia, abdominal and back pain, nausea, vomiting, and diarrhea. HTNV usually causes a more severe form of HFRS than SEOV does.

Shandong Province is located in eastern China and since 1980s, has been one of the areas most seriously affected by HFRS in China (Fang et al., 2010; Huang et al., 2012). Historically, it has had the largest HFRS burden in China–the cumulative number of human cases in Shandong Province accounts for one third of the national total (Kang et al., 2001; Fang et al., 2010). Although the number of human cases has been rapidly reduced within recent decades, it has remained at about 1,000–2,000 cases annually in Shandong, accounting for nearly one fifth of the total number of cases in China. An epidemiologic investigation suggested the presence of two pathogenic species of hantaviruses, HTNV and SEOV in Shandong Province (Li et al., 2007). However, little sequence information on hantaviruses from Shandong has been available to date, and there is limited knowledge on the spatio-temporal epidemiology and genetic characteristics of hantaviruses in Shandong Province. In this study, we conducted an epizootiologic survey, and phylogeographic and phylodynamic analyses to infer the viral phylogenetic relationships in space and time, and gain further insights into the evolutionary dynamics of hantaviruses in Shandong Province, China.

## Materials and methods

### Ethics statement

This study was reviewed and approved by the Ethical Review Board, Science and Technology Supervisory Committee at the Beijing Institute of Microbiology and Epidemiology. The animal work described here adhered to the guidelines of the Animal Subjects Research Review Boards in the Beijing Institute of Microbiology and Epidemiology.

### Sample collection and processing

**Figure 1:**
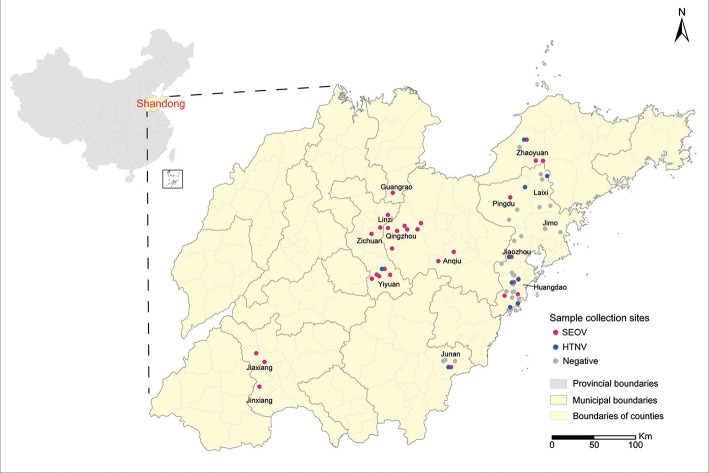
Sample collection sites. Sample collection sites are represented by dots colored according to the disease etiology (red, Seoul virus; blue, Hantaan virus; gray, negative). Map was obtained from the Chinese Academy of Surveying & Mapping. Geographic coordinates of each sample collection site were obtained using the Baidu Coordinates Pick-up System, and were indicated on the map using the ArcGIS 10.2 software.

Rodents were captured in farm lands, grass lands, orchards, and residential areas from 2012 to 2015. Sample collection sites were indicated on a digital map of Shandong Province, which was provided by the Chinese Academy of Surveying & Mapping, using the ArcGIS 10.2 software (ESRI Inc., Redlands, CA, USA) (Figure 1). Cages with a treadle release mechanism were used for live trapping, according to previously described protocols (Mills et al., 1995). All animals were anesthetized with ether, and then sacrificed via cervical dislocation in the field owing to difficulties in transporting them. After the rodent species were identified by morphological assessment, they were necropsied on-site and lung tissues were collected. Instruments that had been cleaned with ethanol and heat-sterilized were used for each animal. Precautions for working with animals potentially infected with dangerous pathogens were strictly followed, and all processes were conducted in accordance with the National Monitoring Program for Hemorrhagic Fever with Renal Syndrome (Trial). Lung tissues were stored either in RNAfixer (Bio Teke Co., China) or liquid nitrogen. After they were transported to the laboratory, tissues were stored at −80°C freezer until further analysis.

Lung tissue samples were screened using the reverse transcription-polymerase chain reaction (RT-PCR). Total RNA, which was extracted from 20 to 50 mg of lung tissue using the TRIzol reagent (Invitrogen, USA), was reverse-transcribed using M-MLV reverse transcriptase (Invitrogen, USA) and the primer P14: 5′-TAGTAGTAGACTCC-3′ (Wang et al., 2000). The PCR was performed to amplify a partial L sequence, using primer pairs as previously described (Klempa et al., 2006). The primers for S and M sequences were designed from consensus regions of SEOV and HTNV sequences (Table 1). The amplicons were sequenced using the ABI 3730XL Genetic Analyzer. The complete M and S sequences were generated from overlapping fragments using the Lasergene package (DNAstar, Inc., USA) and manually edited.

**Table 1:** Primers used for nested and heminested PCR.

**Primer**	**Segment**	**Primer sequence^a^ (5′to 3′)**
SF1	S	TAGTAGTAGRCTCCCTAAAGAGC
SR583	S	TGTAATCTCTTCTGCCTTCATGC
SF207	S	ATCAAAAATTGATGARTTGAAGCGC
SR1189	S	TAACAATTATCCTYTGRTCCAGTTG
SF931	S	ATTGAGTCACCATCATCAATATGGC
SR1642	S	GTTGAGGTAGAATGTTGYTGTTAG
SF1274	S	TGATTGATGTTAAAGTGAAGGAAAT
SR	S	TAGTAGTAGTATKCTCCCTAAARAG
HTN-MF1	M	TAGTAGTAGACTCCGCAAAASAAAGC
HTN-MR494	M	TTACATGCATGGATTGGTACRATCAT
HTN-MR240	M	TTATCCATGCTRCAAGARCTTTC
HTN-MF54	M	GTGGCTAGTAATGGCCAGTTTAGT
HTN-MR2337	M	TACAACCCCAACTYGTCTCATAYTG
HTN-MF107	M	TATGACATGAAAATTGAGTGCCCCCA
HTN-MR892	M	TTGCATTGGCAGGTCCAACWATAGA
HTN-MF591	M	AAATGTTTTGTTCCAGAYCRRAGTGT
HTN-MR1455	M	ACAGCAATDGAATGTGCWACYCCAGG
HTN-MF1203	M	CATGTGAGGCATTTTCTGAAGG
HTN-MR1686	M	TTACAGACATCACAYACCATTG
HTN-MF1428	M	TACCTGGGGTAGCACAYTCWATTGC
HTN-MF2009	M	TGGAATGATAATGCCCATGGRGT
HTN-MR3297	M	CACACTGNGGTGCTCCATCATCAWA
HTN-MF2618	M	AAACATTGGTGYACATCYACATGYCA
HTN-MR2868	M	CTTTCCACTCTATYCKCTCATCAGT
HTN-MF2975	M	ATTGAAGGSGCATGGGGTTCWGGTG
HTN-MR	M	TAGTAGTAGACTCCGCAAGATGTTA
HTN-MF3106	M	TGTTATGGTGCAGAGAGTGTRACA
SEO-MF1	M	TAGTAGTAGACTCCGMAAGARAMAGC
SEO-MR897	M	CCAGGAAGGTCATGRTCTTCYCC
SEO-MR595	M	ACAAAACAYTTYCCTTCTGTCARTAT
SEO-MF484	M	TACATGCCTGCAACATGATGAAAAG
SEO-MR2232	M	CCCAACTATTTTCATACTCATAATC
SEO-MR1209	M	TCACADGATGCACCTGGYCCTGATA
SEO-MF1187	M	TATCAGGRCCAGGTGCATCHTGTGA
SEO-MR1835	M	CAACACCRTGWGYATTATCTGYCCA
SEO-MF2010	M	TGGACAGATAATGCTCAYGGYRTYGG
SEO-MR3291	M	GGWGCACCATCATCATADACTTTYTC
SEO-MR3094	M	CACCATAACAGATTGCCATRTCACA
SEO-MF2796	M	GCAACAATTGATTCTTTCCA
SEO-MF2995	M	GTGACATGGCAATTTGTTATGGTGC
SEO-MR	M	TAGTAGTAGACTCSGMARRWTGT
SEO-MF3144	M	GAAAAGGTGGYCAYAGTGGBTCTT

a *R = A or G; Y = C or T; M = A or C; S = G or C; K = G or T; W = A or T; H = A, C or T; B = C, G or T; D = A, G or T; N = A, G, C, or T*.

### Sequence comparison and phylogenetic analyses

Sequences were compared by the Lasergene package (DNAstar, Inc., USA) to determine the divergence of nucleotide and amino acid sequences.

The sequences were aligned using the Clustalx 1.8 software (Thompson et al., 1994). Phylogenetic trees were generated by the Bayesian Markov Chain Monte Carlo (MCMC) tree-sampling methods, implemented by the MrBayes 3.1 software (Ronquist and Huelsenbeck, 2003) using the GTR evolutionary model, with gamma-distributed rate variation across sites and a proportion of invariable sites. The run was stopped when the standard deviation of split frequencies was below 0.01. The tree was visualized using the FigTree v.1.4.1 software (http://tree.bio.ed.ac.uk/software/figtree/).

To gain a better understanding of the characteristics of the hantaviruses in Shandong Province, we reconstructed the phylogenetic relationships of SEOV and HTNV, including only those strains obtained in the province, based on M and S segment open reading frame (ORF) sequences using two different methods. Maximum clade credibility (MCC) trees were constructed by the Beast v 2.4.6 software (Bouckaert et al., 2014), using the model derived from the Modeltest 0.1 program (Posada, 2008) with the strict clock, relaxed clock log normal, and random local clock models. The convergence was assessed on the basis of the effective sampling size (ESS) using the Tracer v1.6 software (http://tree.bio.ed.ac.uk/software/tracer/). Only log-likelihoods with ESS values of > 200 were accepted. Models were compared by AICM values (Baele et al., 2012), and the lower AICM value was selected as the better model fit. The MCC phylogenetic tree was summarized by the TreeAnnotator program and visualized using the FigTree v1.4.1 program. Phylogenetic network analysis was performed using the median-joining (MJ) method with mutation codons. It was implemented by the Network 5.0.0 software (Bandelt et al., 1999) (http://www.fluxus-engineering.com/sharenet.htm) and invariant sites were removed from the dataset.

### Phylogeny-trait association analyses

The effect of geographic origin on hantavirus populations was evaluated by phylogeny-trait association analysis using the BaTS 2.0 software (Parker et al., 2008), based on M and S segment ORF sequences of SEOV and HTNV. Each BaTS analysis was performed using 180 Bayesian phylogenies, which were obtained using the LogCombiner program. The BaTS analysis of SEOV was run using three states (East, central and Jiaxiang). As for HTNV, we used four states (Northeast, Yiyuan, Jiaozhou and Huangdao). These states were determined according to the phylogenetic trees and geographic distribution of the strains. The BaTS results were reported with three scores: the association index (AI), parsimony score (PS), and monophyletic clade (MC). The AI and PS scores are two methods of testing the overall structure of all traits tested to tree topologies, whereas the MC scores indicate the association of specific traits with the tree topologies. Any score < 0.05 indicated an association for that particular test and score < 0.01 was indicative of strong association.

### Molecular diversity and demographic analyses

The nucleotide diversity (π) and Tajima's *D* test (Tajima, 1989) of the M and S segment ORF sequences for each geographical population were evaluated using the DnaSp 5.0 software (Librado and Rozas, 2009). A value of Tajima's D < 0 means that the population size is not at equilibrium, but expanding, due to an over representation of infrequent polymorphisms, which is typical after a bottleneck or a selective sweep and/or purifying selection.

The Star Contraction (SC) method (Forster et al., 2001) was used to calculate the SC network on the basis of the MJ network for SEOV. The SC network graphs were then compared to those of the MJ network, as this method can facilitate the analysis of historical demographic expansion events. We did not calculate the SC network for HTNV because of its relatively small dataset.

The coding regions of the M and S segments of each strain were concatenated in the order M–S. The population dynamics of hantaviruses were examined using the Bayesian skyline plot (BSP) (Drummond et al., 2005), as implemented in the BEAST program (Bouckaert et al., 2014) with the concatenated sequences. This method is a nonparametric coalescence analysis that uses standard MCMC sampling procedures to estimate past population dynamics from the posterior distribution of the effective population size (Neτ) (Drummond et al., 2002). The model estimation and selection were similar to that of the MCC tree construction.

### Natural selection analyses

The Codon-based *Z* test of selection was performed using the MEGA 7.0 software (Kumar et al., 2016) to test the hypothesis the following about *H0*: (a) *dN* = *dS* (test of neutrality); (b) *dN* > *dS* (positive selection); and (c) *dN* < *dS* (purifying selection). Site-specific selection pressure was assessed using five varying methods, including single likelihood ancestor counting (SLAC), fixed effects likelihood (FEL), internal fixed effects likelihood (IFEL), random effects likelihood (REL), mixed effects model evolution (MEME), and fast unconstrained Bayesian approximation (FUBAR), using the web-based interface Datamonkey (http://www.datamonkey.org/). Significance levels were set to *p* < 0.05, Bayes Factor (BF) > 100, and Posterior Probability (PP) > 0.95.

## Results

### Rodent screening and sequence comparison

A total of 1,798 small animals representing 10 species were captured at 58 different collection sites in Shandong Province (Figure 1). They included *R. norvegicus* (*n* = 908), *A. agrarius* (*n* = 269), *Mus musculus* (*n* = 373), *Sorex araneus* (*n* = 73), *Cricetulus triton* de *Winton* (*n* = 31), *Cricetulus Barabensis* (*n* = 3) *Niviventer confucianus* (*n* = 25), *Rattus flavipectus* (*n* = 23), *Cricetulus spp*. (*n* = 92) and *Microtus arvalis* (*n* = 1). Among them, 111 samples generated the PCR products of expected size for the partial L sequence. Prevalence in individual counties ranged from 0 to 14.3%, and the highest prevalence was found in Qingzhou County (Table 2). Sequence comparison of the PCR products indicated that 93 strains from *R. norvegicus* and one from *M. musculus* were most related to SEOV, with 81.2–98.8% nucleotide similarity. Among them, 93 strains were similar to each other with more than 95.7% nucleotide similarity, and only one strain from *R. norvegicus* in Jiaxiang County (JX20141175) was divergent from most of the SEOV samples obtained in this study with < 83.3% nucleotide similarity. Seventeen samples from *A. agrarius* were similar to the HTNV with 80.9–85.6% nucleotide similarity.

**Table 2:** RT-PCR detection of hantaviruses RNAs in rodents of Shandong Province.

**Areas**	**Period**	**Hosts**				
		***Rattus norvegicus***	***Apodemus agrarius***	***Mus musculus***	**Others**	**Total**
Anqiu	2013–2015	2/20	0/25	0/0	0/0	2/45
Yiyuan	2012–2015	11/108	2/38	0/5	0/4	13/155
Zichuan	2015	4/22	0/2	0/1	0/2	4/27
Huangdao	2012–2015	11/159	6/69	0/124	0/128	17//480
Zhaoyuan	2013–2014	3/105	3/6	0/15	0/8	6/134
Jiaxiang	2013–2014	24/242	0/18	0/34	0/42	24/336
Pingdu	2015	1/19	1/2	0/8	0/1	2/30
Qingzhou	2014–2015	25/102	0/29	1/33	0/18	26/182
Jiaozhou	2013–2015	0/11	3/25	0/66	0/13	3/115
Laixi	2015	0/25	1/22	0/24	0/4	1/75
Linzi	2014	10/67	0/2	0/7	0/2	10/78
Junan	2012–2014	1/17	1/31	0/34	0/25	2/107
Guangrao	2013	1/6	0/0	0/0	0/0	1/6
Jimo	2015	0/5	0/0	0/22	0/1	0/28
Total	2012–2015	93/908	17/269	1/373	0/248	111/1,798

*Data indicate the number of positive for hantavirus RNA/the number of rodents captured*.

A total of 76 complete M segment, and 71 complete S segment nucleotide sequences of SEOV were also determined. They were all obtained from *R. norvegicus*, with the exception of one strain from *M. musculus*. The M sequences of SEOV were 3,644–3,656 nt long. They all consisted of an ORF of 3,402 nt that encoded a putative 1,134 amino acid (aa) glycoprotein precursor with 95.3–99.3% nucleotide similarity to other SEOV strains. The complete S sequences of SEOV were 1,764–1,775 nt long. They all contained the single ORF that encoded the N protein of 430 aa with 95.7–99.4% nucleotide similarity to other SEOV strains. No differences were observed between the SEOV from *R. norvegicus* and that from *M. musculus*. We did not obtain the M and S sequences from the sample JX20141175.

A total of 14 complete M segment, and 13 complete S segment nucleotide sequences of HTNV were determined. The complete M sequences of HTNV were 3,605–3,616 nt long, containing a single ORF that encoded a putative 1,136 aa glycoprotein precursor. They were similar to other HTNV strains with 84.0–96.7% sequence similarity. The complete S sequences of HTNV were 1,701–1,703 nt long. All contained a single ORF that encoded a putative nucleocapsid (N) protein of 430 aa. It showed 85.8–97.0% nucleotide similarity to other HTNV strains.

### Phylogenetic analyses

**Figure 2:**
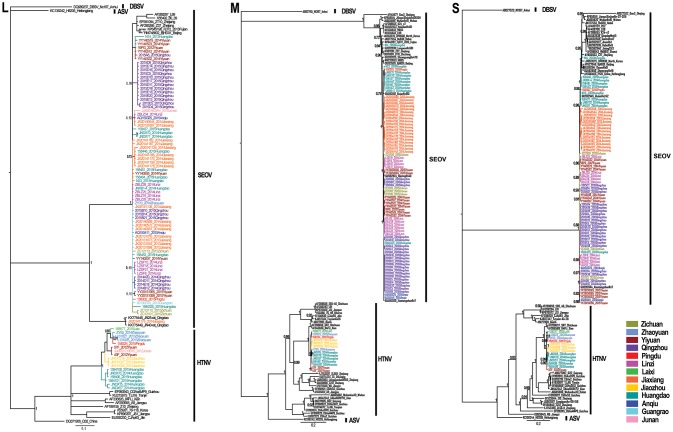
Phylogenetic trees estimated by the Bayesian method using the MrBayes software based on the 329-nucleotide alignment of L-segment sequences, and ORF sequences of the M segment and S segment. Phylogenetic trees were constructed using the GTR evolutionary model, with Dabieshan virus NC167 strain as the outgroup, gamma-distributed rate variation across sites and a proportion of invariable sites. The run was stopped when the standard deviation of split frequencies reached below 0.01. The L (accession no. KY639712–KY639799), M (accession no. KY639536–KY639625), and S (accession no. KY639626–KY639711) sequences of hantaviruses obtained in this study are shown in different colors according to their geographic origin. The numbers at each node are posterior probabilities (PP) based on 6,000,000–12,000,000 trees. Only PP values > 0.6 are shown. The scale bar indicates the nucleotide substitutions per site. SEOV, Seoul virus; HTNV, Hantaan virus; DBSV, Dabieshan virus; ASV, Amur-Soochong virus.

Phylogenetic trees were constructed, including partial L segments (*n* = 94) (accession no. KY639712-KY639799), ORF sequences of M (*n* = 90) (accession no. KY639536-KY639625), and S segments (*n* = 84) (accession no. KY639626-KY639711) obtained in this study, and other representative sequences from different areas in East Asia downloaded from GenBank. Combined with the geographic origin of the strains, the results showed the distribution of SEOV throughout Shandong Province, whereas HTNV seemed to be co-circulating with SEOV only in the eastern areas (Zhaoyuan, Laixi, Pingdu, Jiaozhou, and Huangdao) and southern areas (Junan and Yiyuan) (Figures 1, 2). The phylogenetic trees indicated that most SEOV strains from Shandong clustered together with the strains from other areas in China, and the genetic variants from a single collection site clustered together (Figure 2). Only one strain from Jiaxiang (JX20141175) clustered together with the strains from Qingdao and formed a new lineage of SEOV in the L tree. They seemed to be a new subtype of SEOV. The HTNV strains from Shandong were clustered together with other HTNV strains and formed a new sister lineage (Figure 2). The phylogenetic trees also indicated geographic clustering of both HTNV and SEOV, especially for the M and S segments. This might be due to the increased length of the M and S sequences in the present study, which could have provided more information.

**Figure 3:**
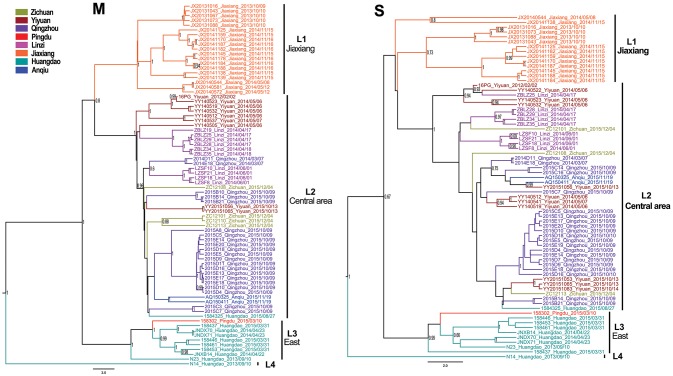
Relative phylogenetic relationships of M and S gene sequences for Seoul virus defined in MCC trees. Different colors represent the geographic origin of the samples. Numbers at each node are posterior probabilities (PP) based on 6,000,000–12,000,000 trees. Only PP values > 0.6 are shown. The scale bar indicates the nucleotide substitutions per site.

**Figure 4:**
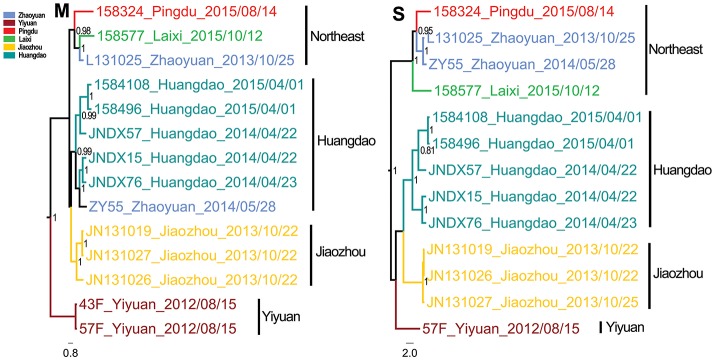
Relative phylogenetic relationships of M and S segment sequences for Hantaan virus defined in MCC trees. Different colors represent the geographic origin of the samples. Numbers at each node are posterior probabilities (PP) based on 1,000,000–5,000,000 trees. Only PP values > 0.6 are shown. The scale bar indicates the nucleotide substitutions per site.

The Bayesian MCC trees of SEOV indicated that the strains from Shandong fell into four different lineages for both M and S segments (Figure 3), which were roughly consistent with their geographic origin. Lineage 1 included the strains from Jiaxiang County. Lineage 2 included the strains from the central areas (Linzi, Zichuan, Qingzhou, Yiyuan, and Anqiu), and one strain (1584325) obtained in Huangdao. Lineage 3 included strains from the eastern areas (Huangdao and Pingdu). Lineage 4 included only one strain from Huangdao. The MCC trees of HTNV strains in Shandong showed that they fell into four different lineages according to their geographic distribution (Figure 4), specifically, the strains from Huangdao, Yiyuan, Jiaozhou, and strains from the northeastern areas (Zhaoyuan, Laixi, and Pingdu). Interestingly, the strain ZY55 fell within the Huangdao cluster for the M-segment, and within the Northeastern cluster for the S segment. However, the notion that the ZY55 strain was a reassortment was not strongly supported by the present data, as the PP value for the stem was very low (0.44) in the M phylogenetic tree.

**Figure 5:**
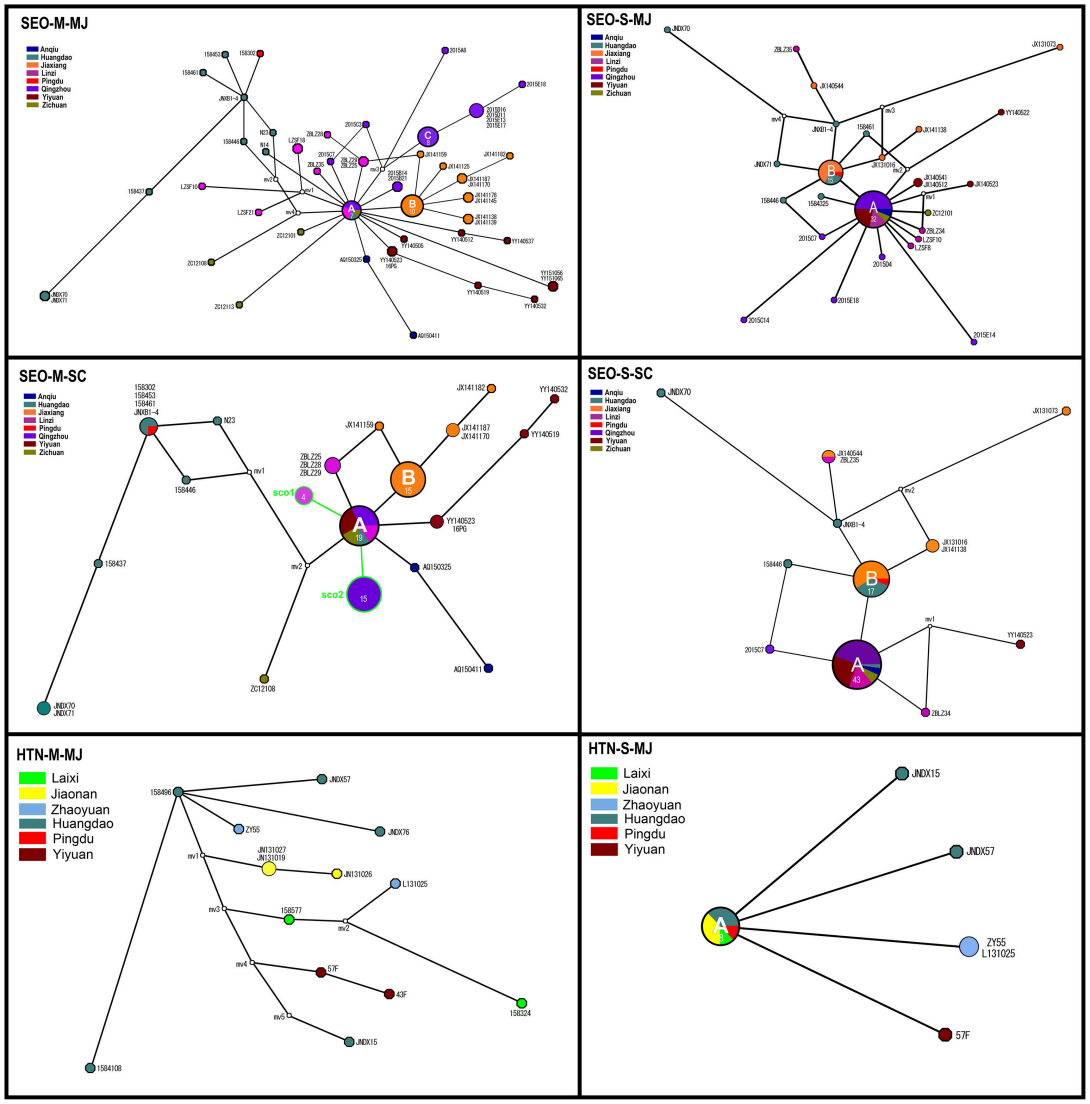
Median-joining (MJ) and Star Contraction (SC) phylogenetic networks of Seoul virus and Hantaan virus. Each pie chart was colored according to the geographic origin of the samples and sized relative to its frequency in the dataset. The numbers in each pie chart represent the haplotypes of the strains. Small white circles represent median vectors (roughly equivalent to hypothetical unsampled haplotypes). Length of the branch is proportional to the number of mutational changes between haplotypes.

The MJ network, constructed from 46 alleles for the M segment, demonstrated that three haplotypes (A, B, and C) were highly common and shared by various strains (Figure 5, SEO-M-MJ). Haplotype A included strains from the central areas, and was connected to several low-frequency tip haplotypes from those areas. This suggested that most strains in these areas shared the same ancestor. Haplotype B mainly included strains from Jiaxiang. Haplotype C included strains from Qingzhou County. The MJ network, constructed from 30 alleles for S segment of SEOV, indicated that only two haplotypes (A and B) were highly common and similar to those in SEO-M-MJ (Figure 5, SEO-S-MJ). The MJ network of HTNV showed no obvious haplotype trait.

### Phylogeny-trait association analysis

Phylogeny-trait association analysis using the BaTS 2.0 software detected a very strong association between the geographic traits and phylogeny for both SEOV and HTNV (*p* = 0 for AI and PS statistics). When the MC statistic was used to test the extent of the phylogenetic clustering of SEOV in three regions (East, Central, and Jiaxiang), the results showed high significance (*p* < 0.01) among these geographic populations (Table 3). The MC statistic for HTNV (four regions) showed no significance in some populations (*p* > 0.05) (Table 3). Nevertheless, it should be noted that only a limited number of HTNV sequences were available in the present study, which might have led to false evaluation.

**Table 3:** Region-phylogeny association parameters of hantaviruses in Shandong Province (three states for SEOV and four states for HTNV).

**Hantaviruses**	**Segment**	**Statistic**	**Observed mean**	**Lower 95% CI**	**Upper 95% CI**	**Null mean**	**Lower 95% CI**	**Upper 95% CI**	**Significance**
SEOV	M	AI	0.0348	0.0000	0.1020	4.8757	4.0315	5.7825	0.0000^**^
		PS	3.0000	3.0000	3.0000	27.0148	24.6556	29.2556	0.0000^**^
		MC (East)	9.0000	9.0000	9.0000	1.5021	1.0000	2.7944	0.0099^**^
		MC (Central)	19.9722	14.0000	27.0000	4.3408	3.0889	5.6222	0.0099^**^
		MC (Jiaxiang)	19.0000	19.0000	19.0000	2.0427	1.1611	3.0000	0.0099^**^
	S	AI	0.0439	0.0000	0.2500	4.2619	3.5325	4.9381	0.0000^**^
		PS	3.0778	3.0000	4.0000	22.9948	21.4222	24.3889	0.0000^**^
		MC (East)	9.5556	9.0000	10.0000	1.4853	1.0000	2.0556	0.0099^**^
		MC (Central)	19.6333	14.0000	28.0000	4.7687	3.5778	7.6111	0.0099^**^
		MC (Jiaxiang)	13.7778	13.0000	14.0000	1.7432	1.0667	2.4111	0.0099^**^
HTNV	M	AI	0.0919	0.0906	0.1045	1.4889	0.9315	1.8570	0.0000^**^
		PS	3.9722	4.0000	4.0000	7.8678	6.7500	9.0000	0.0000^**^
		MC (Northwest)	3.0000	3.0000	3.0000	1.3094	1.0000	2.0000	**0.0299**
		MC (Huangdao)	3.0556	3.0000	3.0000	1.5597	1.0000	3.0000	0.0699
		MC (Yiyuan)	2.0000	2.0000	2.0000	1.0900	1.0000	2.0000	0.1000
		MC (Jiaozhou)	3.0000	3.0000	3.0000	1.1400	1.0000	2.0000	**0.0299**
	S	AI	0.0061	0.0032	0.0154	1.2678	0.8761	1.6336	0.0000^**^
		PS	3.0000	3.0000	3.0000	6.9759	5.6500	8.0000	0.0000^**^
		MC (Northwest)	4.0000	4.0000	4.0000	1.3720	1.0000	2.0167	0.0099^**^
		MC (Huangdao)	5.0000	5.0000	5.0000	1.5048	1.0000	2.8944	0.0099^**^
		MC (Yiyuan)	1.0000	1.0000	1.0000	1.0000	1.0000	1.0000	1.0000
		MC (Jiaozhou)	3.0000	3.0000	3.0000	1.2137	1.0000	2.0722	0.0099^**^

*Bold values were significant at p < 0.05*,

** *indicates significance at p < 0.01*.

*CI, confidence interval; AI, association index; PS, parsimony score; MC, monophyletic clade; SEOV, Seoul virus; HTNV, Hantaan virus*.

### Molecular diversity and demographic analysis

Neutrality tests performed on the M and S datasets produced negative Tajima's D values for most of the geographic populations (Table 4). Particularly in the central areas, the Tajima's *D* test value was significant for SEOV, which might be the main reason that these test results were significant for “all areas.” These findings support a model of the population expansion of SEOV in the central areas. In addition, when Tajima's *D* test was performed, multiple evolutionary paths were detected for the M segment of SEOV in the eastern areas (Huangdao and Pingdu), and for both M and S segments in the central areas.

**Table 4:** Genetic diversity indices and demographic history parameters of hantaviruses in Shandong Province.

**Hantaviruses**	**Areas**	**M segment**		**S segment**	
		**Nucleotide diversity (π)**	**Tajima's D**	**Nucleotide diversity (π)**	**Tajima's D**
SEOV	East (Huangdao, Pingdu)	0.0145	−**1.3920**	0.0113	**–**1.2630
	Central areas (Yiyuan, Linzi, Zichuan, Anqiu and Qingzhou)	0.0085	−1.8665^*^	0.0070	−2.0773^*^
	Jiaxiang	0.0076	0.3759	0.0090	**–**0.8371
	All	0.0135	−1.8196^*^	0.0104	−2.1217^*^
HTNV	Northeast (Laixi, Pingdu and Zhaoyuan)	0.0115	**–**0.5765	0.0071	**–**0.1167
	South (Jiaozhou, Yiyuan and Huangdao)	0.0109	**–**0.2857	0.0095	**–**0.1271
	All	0.0123	**–**0.6944	0.0133	**–**0.3130

* *Indicates significance at p < 0.05*.

*Bold values indicate that some codons with multiple evolutionary paths were detected when Tajima's D test was performed*.

*SEOV, Seoul virus; HTNV, Hantaan virus*.

Historical demographic expansion events are characterized by star-like clusters of nodes around a founder population node. The SC algorithm can identify such clusters and shrink the nodes of a cluster back toward the founder node. We constructed the SC network and compared it with the MJ network to facilitate the analysis of the historical demographic expansion events. The results indicated two founder nodes (sco1 and sco2) for the M segment (Figure 5, SEO-M-SC). The sco1 node was formed by contraction of the strains from Linzi. The sco2 node was formed by strains from Qingzhou which were contracted within haplotype C. In addition, some strains from the central areas were contracted within haplotype A, and some strains from Jiaxiang were contracted within haplotype B (Figure 5, SEO-M-SC). Although no founder nodes were detected in the SC network of the S segment, some strains from the central areas were contracted within haplotype A (Figure 5, SEO-S-SC). These results suggest that there might be historical demographic expansion events for SEOV in the central areas. These findings are consistent with Tajima's *D* test results.

**Figure 6:**
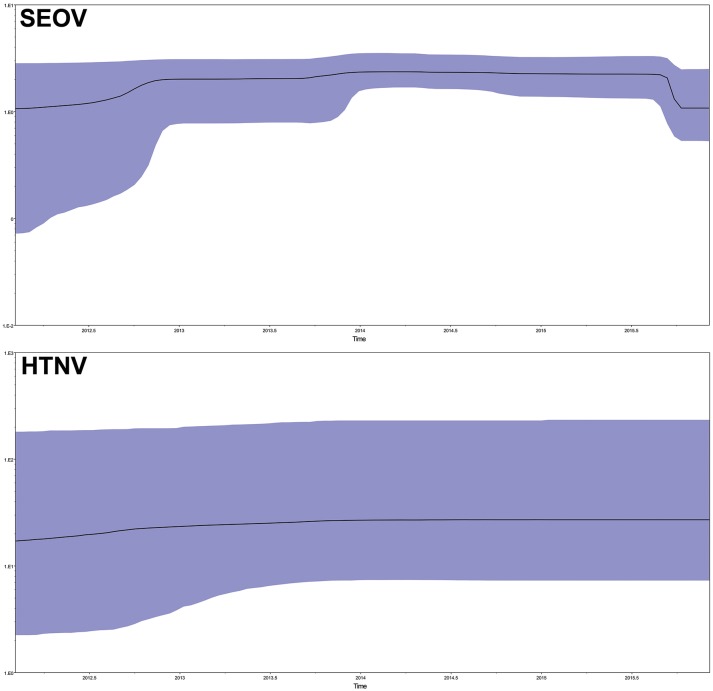
Population dynamics of SEOV and HTNV in Bayesian skyline reconstruction plots. The demographic inference of SEOV and HTNV genetic diversity is depicted using the Bayesian skyline reconstruction plot method. The plot was referred to the concatenated sequences in the order M–S. Solid lines indicate the median estimates, and the purple area displays the 95% highest posterior density (HPD). SEOV, Seoul virus; HTNV, Hantaan virus.

To explore the demographic history of the sampled population over the study period, the relative effective population size over time was inferred using the Bayesian skyline model, which essentially plots effective population size (Neτ) as a function of time. The results indicated that the effective population size of SEOV increased before the end of 2012, reached a plateau (2013–2015), and then rapidly decreased (Figure 6, SEO). The HTNV population remained roughly steady (Figure 6, HTN).

### Selection pressure analyses

The selection pressure analyses using the *Z* test and codon-specific analyses revealed that negative selection was acting as the principal evolutionary force, with very low *dN*/*dS* values (far lower than 1), and an abundance of negatively selected sites (Table 5). The mean *dN*/*dS* values for Gc was 0.0393 and the *dN*/*dS* values for Gn was 0.0669. Codon-specific analyses also indicated little evidence of positive selection (Table 5). One positively selected site in the Gn/Gc protein and one in the N protein of SEOV showed significance (*p* < 0.05), based on the MEME method. Another positively selected site was found in the N protein of SEOV using the FUBAR method yielding a significance value (*p* < 0.05). Regarding the Gn/Gc protein of HTNV, three positively selected sites based on the REL method, and another positively selected site based on the FUBAR method all yielded a significance value (*p* < 0.05). No positively selected sites were detected in the N protein of HTNV.

**Table 5:** Summary of selection pressures acting on Gn/Gc protein and N protein of SEOV and HTNV obtained in this study.

**Protein**	**Method**	**SEOV**			**HTNV**		
		**dN/dS**	**Position of diversifying codons (*p* < 0.05 or BF > 100 or PP > 0.95)**	**Position of Purifying selected codons (*p* < 0.05 or BF > 100 or PP > 0.95)**	**dN/dS**	**Position of diversifying codons (*p* < 0.05 or BF > 100 or PP > 0.95)**	**Position of Purifying selected codons (*p* < 0.05 or BF > 100 or PP > 0.95)**
Gn/Gc	SLAC	0.0462	0	39 codons	0.0617	0	7 codons
	FEL	–	0	82 codons	–	0	26 codons
	IFEL	–	0	36 codons	–	0	16 codons
	MEME	–	1 codon: 827	–	–	1 codon: 30	–
	REL	–	0	1134 codons	–	3 codons: 289, 373, 889	7 codons
	FUBAR	–	0	313 codons	–	0	29 codons
N	SLAC	0.0962	0	7 codons	0.0311	0	0
	FEL	–	0	27 codons	–	0	4 codons
	IFEL	–	0	3 codons	–	0	1 codon
	MEME	–	1 codon: 37	–	–	0	–
	REL	–	0	129 codons	–	0	50 codons
	FUBAR	–	1 codon: 367	30 codons	–	0	5 codons

*SEOV, Seoul virus; HTNV, Hantaan virus; SLAC, single likelihood ancestor counting; FEL, fixed effects likelihood; IFEL, internal fixed effects likelihood; MEME, mixed effects model evolution; REL, random effects likelihood; FUBAR, unconstrained bayesian approximation; BF, Bayes factors (REL); PP, posterior probability (FUBAR)*.

## Discussion

In this study, we carried out an epizootiological investigation by collecting a large number of samples in 58 collection sites, covering the most severe affected HFRS endemic areas in Shandong Province, China (Fang et al., 2010; Wei et al., 2011; Cui et al., 2013; Wang et al., 2016). We found that both SEOV and HTNV were co-circulating in eastern and southern Shandong. However, the dominant hantavirus in the western and northern parts of Shandong was SEOV. Generally, HTNV is mainly associated with *A. agrarius*, which remains predominant in rural and forested areas, whereas SEOV is typically carried by *R. norvegicus*, which is distributed in almost all areas, especially the urban areas. Our results suggest that prioritization of control efforts should be focused on peridomestic rodents, such as *R. norvegicus* in residential areas throughout Shandong Province. *M. musculus* should also be monitored, as they could be affected by spillover infections with SEOV. However, the virus seems to be far less prevalent in *M. musculus* than in *R. norvegicus*. In eastern and southern Shandong, both the sylvatic and peridomestic rodents, *R. norvegicus* and *A. agrarius* should be monitored.

Phylogenetic analysis indicated that one strain from Jiaxiang (JX20141175) clustered together with two strains from Qingdao and formed a new lineage of SEOV in the L tree. This seemed to be a new subtype of SEOV that was widely distributed in Shandong. Unfortunately, we did not obtain the M and S sequences of the sample because of the high level of nucleotide divergence and limited availability of the tissue. Furthermore, no information on the M and S sequences of these strains was available in GenBank. Therefore, further epidemiological and virological studies in Shandong Province are necessary to evaluate the issue further.

Phylogeny-trait association analyses indicated very strong clustering of SEOV by geographic origin. This suggests that SEOV could be transmitted over a limited distance under normal conditions, possibly because its spread was accompanied by dispersion of the reservoir, which usually moves within a limited range. Therefore, the dispersal of SEOV should be slow and steady under wild conditions. However, when Tajima's *D* test was performed, some codons with multiple evolutionary paths were detected in the central areas. In addition, significant negative Tajima's *D* test value supported a model of population expansion for SEOV in the central areas. The SC analyses also suggested historical demographic expansion events associated with SEOV in the central areas. Furthermore, Bayesian skyline plot suggested an increase of the effective population size of SEOV. These results all suggest that SEOV in the central areas might have diverse origins and they might have experienced rapid and wide spread. This phenomenon was possibly related to its animal host, *R. Norvegicus*, which is spatially in closer proximity to human beings than any other hosts of hantaviruses. These hosts could migrate to other places further away by means of transportation. Therefore, SEOV could be transmitted over a relatively long distance by infected *R. Norvegicus* aboard motor vehicles (Plyusnin and Morzunov, 2001; Zuo et al., 2011). In addition, SEOV could be spread over a long distance via the fecal matter of infected *R. norvegicus* on cargo. These factors could facilitate the long-distance transmission of SEOV, which might be responsible for the appearance of SEOV in Europe (Plyusnina et al., 2012; Dupinay et al., 2014; Reynes et al., 2017). These factors also created favorable conditions for SEOV from different origins to enter the same area. A Combination of the highly frequent cargo trade in Shandong Province, with the construction of several highways likely presents suitable conditions for the transmission and dispersal of, and interaction between, SEOV strains, which is considered to be ongoing. Therefore, both SEOV and its hosts, *R. Norvegicus*, should be rigorously monitored, in order to control the disease in Shandong Province, particularly in freight terminals, wharfages and warehouses.

Our study indicates that hantaviruses in Huangdao show the greatest diversity, with two species of hantaviruses, and the highest nucleotide diversity for SEOV (0.0141 for the M segment and 0.0107 for the S segment) in Shandong. The present study also indicates that some codons with multiple evolutionary paths were detected for the M segment of SEOV in Huangdao when Tajima's *D* test was performed. Interestingly, the strain 1584325 from Huangdao shared the same ancestor with strains from the central areas. This finding suggests that this strain might have been transmitted from the central areas and could be regarded as an example of long-distance transmission of SEOV. Hantavirus diversity and complexity in the Huangdao district might be related to its geographical position. Huangdao is one of the emerging economic development zones located in Qingdao City, a port city with heavy traffic congestion. The city is affected by modes of transport over land and sea that move imports and exports on a daily basis. In addition, hantaviruses in this area seem to have undergone a relatively long evolutionary history, as a previous study indicated that Huangdao was one of the first locations at which hantaviruses appeared in Shandong (Fang et al., 2010). All of these factors increased the chances of the transmission of, and interactions among, the various strains of hantaviruses.

Understanding the driving forces acting on viral evolution and its associated mechanisms is one of central aspects of evolutionary biology. In addition, the study of these forces would facilitate the development of efficient strategies for the management of viral diseases. In this study, several methods of selection pressure analyses showed that the ratio of non-synonymous to synonymous substitutions for Gn/Gc and the N protein gene was very low, indicating that most amino acid changes were deleterious polymorphisms removed by negative selection. In comparison to the Gc gene sequence, the greater non-synonymous Gn sequence variation was consistent with membrane-distal localization and supported the notion that Gn was subjected to selective pressure of the humoral immune response (Li et al., 2016). Previous studies have also suggested that negative selection acts as the principal evolutionary force on SEOV and HTNV (Hughes and Friedman, 2000; Lin et al., 2012; Castel et al., 2014). Negative selection has important implications for the evolution of RNA viruses, because a large number of rare non-synonymous mutations resulting from viral replication are often purified from the population (Hughes and Hughes, 2007).

Based on our analyses, in addition to negative selection pressure, episodic diversifying selection could be another evolutionary force working on the hantavirus genomes, even though several sites were positively selected. We identified one positively selected site at aa 827 of the Gn/Gc protein, and two positively selected sites at aa 37 and aa 367 of the N protein for SEOV. The aa 35–38 of the N protein reportedly shows strong cross reactivity with, and is important for the recognition of, the SEOV antigen (Lindkvist et al., 2008). We identified three positively selected sites for the Gn/Gc protein of HTNV at aa 289, aa 373, and aa 889. The aa 882–896 is reportedly a B-cell epitope that displays neutralizing activity to HTNV infection (Yan et al., 2012). The higher proportion of positively selected positions located in the variable Gn/Gc envelope glycoproteins was consistent with their functional roles in the viral escape from immunological responses (Holmes, 2003). We noticed that none of these positively selected sites were identified by more than one method. Although the accurate functions of all mutated sites remain unknown, based on the literature, some evidently play important functional roles. Other positively selected sites might also have important functional roles. However, these findings do not necessarily mean that identification by one method was sufficient to conclude whether a site was indeed under positive selection. The natural selection profiles of the various sites should be further tested. We presumed that the mutated sites might provide a selective advantage for the virus to evade host immunity, and indicate the importance of the associated residues for the adaptive evolution of hantaviruses. These findings might be beneficial to the development of vaccines.

## Author contributions

S-QZ, X-JL, Z-QW, and W-CC conceived and designed the experiments. S-QZ, X-JL, Z-QW, and J-FJ collected the samples. S-QZ, X-JL, J-SZ, and Q-MZ performed the experiments. S-QZ, L-QF, and W-HZ analyzed the data. S-QZ, X-JL, and W-CC wrote the paper. All authors reviewed the manuscript.

### Conflict of interest statement

The authors declare that the research was conducted in the absence of any commercial or financial relationships that could be construed as a potential conflict of interest.

## References

[B1] Baele G. Lemey P. Bedford T. Rambaut A. Suchard M. A. Alekseyenko A. V. (2012). Improving the accuracy of demographic and molecular model clock model comparison while accommodating phylogenetic uncertainty. Mol. Biol. Evol. 29, 2157–2167. 10.1093/molbev/mss084 22403239 PMC3424409

[B2] Bandelt H. J. Forster P. Röhl A. (1999). Median-joining networks for inferring intraspecific phylogenies. Mol. Biol. Evol. 16, 37–48. 10.1093/oxfordjournals.molbev.a026036 10331250

[B3] Bouckaert R. Heled J. Kühnert D. Vaughan T. Wu C. H. Xie D.. (2014). BEAST 2: a software platform for Bayesian evolutionary analysis. PLoS Comput. Biol. 10:e1003537. 10.1371/journal.pcbi.1003537 24722319 PMC3985171

[B4] Castel G. Razzauti M. Jousselin E. Kergoat G. J. Cosson J. F. (2014). Changes in diversification patterns and signatures of selection during the evolution of murinae-associated hantaviruses. Viruses 6, 1112–1134. 10.3390/v6031112 24618811 PMC3970142

[B5] Cui F. Wang T. Wang L. Yang S. Zhang L. Cao H.. (2013). Spatial analysis of hemorrhagic fever with renal syndrome in zibo City, China, 2009–2012. PLoS ONE 8:e67490. 10.1371/journal.pone.0067490 23840719 PMC3696076

[B6] Drummond A. J. Nicholls G. K. Rodrigo A. G. Solomon W. (2002). Estimating mutation parameters, population history and genealogy simultaneously from temporally spaced sequence data. Genetics 161, 1307–1320. 12136032 10.1093/genetics/161.3.1307 PMC1462188

[B7] Drummond A. J. Rambaut A. Shapiro B. Pybus O. G. (2005). Bayesian coalescent inference of past population dynamics from molecular sequences. Mol. Biol. Evol. 22, 1185–1192. 10.1093/molbev/msi103 15703244

[B8] Dupinay T. Pounder K. C. Ayral F. Laaberki M. H. Marston D. A. Lacôte S.. (2014). Detection and genetic characterization of seoul virus from commensal brown rats in France. Virol. J. 11:32. 10.1186/1743-422X-11-32 24555484 PMC3944734

[B9] Fang L. Q. Wang X. J. Liang S. Li Y. L. Song S. X. Zhang W. Y.. (2010). Spatiotemporal trends and climatic factors hemorrhagic fever with renal syndrome epidemic in Shandong Province, China. PLoS Negl. Trop. Dis. 4:e789. 10.1371/journal.pntd.0000789 20706629 PMC2919379

[B10] Forster P. Torroni A. Renfrew C. Röhl A. (2001). Phylogenetic star contraction applied to Asian and Papuan mtDNA evolution. Mol. Biol. Evol. 18, 1864–1881. 10.1093/oxfordjournals.molbev.a003728 11557793

[B11] Holmes E. C. (2003). Error thresholds and the constraints to RNA virus evolution. Trends Microbiol. 11, 543–546. 10.1016/j.tim.2003.10.006 14659685 PMC7172642

[B12] Huang X. Yin H. Yan L. Wang X. Wang S. (2012). Epidemiologic characteristics of haemorrhagic fever with renal syndrome in Mainland China from 2006 to 2010. WPSAR 3, 12–18. 10.5365/wpsar.2011.2.2.007 23908902 PMC3729070

[B13] Hughes A. L. Friedman R. (2000). Evolutionary diversification of protein-coding genes of hantaviruses. Mol. Biol. Evol. 17, 1558–1568. 10.1093/oxfordjournals.molbev.a026254 11018161

[B14] Hughes A. L. Hughes M. A. (2007). More effective purifying selection on RNA viruses than in DNA viruses. Gene 404, 117–125. 10.1016/j.gene.2007.09.013 17928171 PMC2756238

[B15] Jiang H. Du H. Wang L. M. Wang P. Z. Bai X. F. (2016). Hemorrhagic fever with renal syndrome: pathogenesis and clinical picture. Front. Cell. Infect. Microbiol. 6:1 10.3389/fcimb.2016.00001 26870699 PMC4737898

[B16] Jonsson C. B. Figueiredo L. T. Vapalahti O. (2010). A global perspective on hantavirus ecology, epidemiology, and disease. Clin. Microbiol. Rev. 23, 412–441. 10.1128/CMR.00062-09 20375360 PMC2863364

[B17] Kang D. M. Ruan Y. H. Fu J. H. Zhang Z. B. Zhang X. L. Wand K. A. (2001). Epidemic characteristic and its changing trend of hemorrhagic fever with renal syndrome in Shandong Province from 1990 to 1998. Chin. J. Epidemiol. 22, 475–476.

[B18] Klempa B. Fichet-Calvet E. Lecompte E. Auste B. Aniskin V. Meisel H.. (2006). Hantavirus in African wood mouse, Guinea. Emerg. Infect. Dis. 12, 838–840. 10.3201/eid1205.051487 16704849 PMC3374458

[B19] Kumar S. Stecher G. Tamura K. (2016). MEGA7: molecular evolutionary genetics is version 7.0 for bigger datasets. Mol. Biol. Evol. 33, 1870–1874. 10.1093/molbev/msw054 27004904 PMC8210823

[B20] Li J. Zhao Z. Wang Z. Liu Y. Hu M. (2007). Nucleotide sequence characterization and phylogenetic is of hantaviruses isolated in Shandong Province, China. Chin. Med. J. 120, 825–830. 17531126

[B21] Li S. Rissanen I. Zeltina A. Hepojoki J. Raghwani J. Harlos K.. (2016). A molecular-level account of the antigenic hantaviral surface. Cell. Rep. 15, 959–967. 10.1016/j.celrep.2016.03.082 27117403 PMC4858563

[B22] Librado P. Rozas J. (2009). DnaSP v5: a software for comprehensive is of DNA polymorphism data. Bioinformatics 25, 1451–1452. 10.1093/bioinformatics/btp187 19346325

[B23] Lin X. D. Wang W. Guo W. P. Zhang X. H. Xing J. G. Chen S. Z.. (2012). Cross-species transmission in the speciation of the currently known murinae-associated hantaviruses. J. Virol. 86, 11171–11182. 10.1128/JVI.00021-12 22855492 PMC3457156

[B24] Lindkvist M. Näslund J. Ahlmb C. Buchta G. (2008). Cross-reactive and serospecific epitopes of nucleocapsid proteins of three hantaviruses: prospects for new diagnostic tools. Virus. Res. 137, 97–105. 10.1016/j.virusres.2008.06.003 18620010

[B25] Mills J. N. Childs J. E. Ksiazek T. G. Peters C. J. Velleca W. M. (1995). Methods for Trapping and Sampling Small Mammals for Virologic Testing. Atlanta, GA: Centers for Disease Control and Prevention.

[B26] Mir M. A. (2010). Hantaviruses. Clin. Lab. Med. 30, 67–91. 10.1016/j.cll.2010.01.004 20513542 PMC2880890

[B27] Parker J. Rambaut A. Pybus O. G. (2008). Correlating viral phenotypes with phylogeny: accounting for phylogenetic uncertainty. Infect. Genet. Evol. 8, 239–246. 10.1016/j.meegid.2007.08.001 17921073

[B28] Plyusnin A. Morzunov S. P. (2001). Virus evolution and genetic diversity of hantaviruses and their rodent hosts. Curr. Top. Microbiol. 256, 47–75. 10.1007/978-3-642-56753-7_4 11217406

[B29] Plyusnina A. Heyman P. Baert K. Stuyck J. Cochez C. Plyusnin A. (2012). Genetic characterization of Seoul hantavirus originated from Norway rats (*Rattus norvegicus*) captured in Belgium. J. Med. Virol. 84, 1298–1303. 10.1002/jmv.23321 22711359

[B30] Posada D. (2008). jModelTest: phylogenetic model averaging. Mol. Biol. Evol. 25, 1253–1256. 10.1093/molbev/msn083 18397919

[B31] Reynes J. M. Carli D. Bour J. B. Boudjeltia S. Dewilde A. Gerbier G.. (2017). Seoul virus infection in Humans, France, 2014–2016. Emerg. Infect. Dis. 23, 973–977. 10.3201/eid2306.160927 28368241 PMC5443425

[B32] Ronquist F. Huelsenbeck J. P. (2003). MrBayes 3: bayesian phylogenetic inference under mixed models. Bioinformatics 19, 1572–1574. 10.1093/bioinformatics/btg180 12912839

[B33] Tajima F. (1989). The effect of change in population size on DNA polymorphism. Genetics 123, 597–601. 2599369 10.1093/genetics/123.3.597 PMC1203832

[B34] Thompson J. D. Higgins D. G. Gibson T. J. (1994). CLUSTAL W: improving the sensitivity of progressive multiple sequence alignment through sequence weighting, position-specific gap penalties and weight matrix choice. Nucleic Acids Res. 22, 4673–4680. 10.1093/nar/22.22.4673 7984417 PMC308517

[B35] Wang H. Yoshimatsu K. Ebihara H. Ogino M. Araki K. Kariwa H.. (2000). Genetic diversity of hantaviruses isolated in china and characterization of novel hantaviruses isolated from niviventer confucianus and *Rattus rattus*. Virology 278, 332–345. 10.1006/viro.2000.0630 11118357

[B36] Wang L. Wang T. Cui F. Zhai S. Y. Zhang L. Yang S. X.. (2016). Hemorrhagic fever with renal syndrome, Zibo City, China, 2006–2014. Emerg. Infect. Dis. 22, 274–276. 10.3201/eid2202.151516 26812444 PMC4734509

[B37] Wei L. Qian Q. Wang Z. Q. Glass G. E. Song S. X. Zhang W. Y.. (2011). Using geographic information system-based ecologic niche models to forecast the risk of hantavirus infection in Shandong Province, China. Am. J. Trop. Med. Hyg. 84, 497–503. 10.4269/ajtmh.2011.10-0314 21363991 PMC3042829

[B38] Yan G. Zhang Y. Ma Y. Yi J. Liu B. Xu Z.. (2012). Identification of a novel B-cell epitope of hantaan virus glycoprotein recognized by neutralizing 3D8 monoclonal antibody. J. Gen. Virol. 93, 2595–2600. 10.1099/vir.0.045302-0 22933664

[B39] Zeier M. Handermann M. Bahr U. Rensch B. Müller S. Kehm R.. (2005). New ecological aspects of hantavirus infection: a change of a paradigm and a challenge of prevention–a review. Virus Genes 30, 157–180. 10.1007/s11262-004-5625-2 15744574

[B40] Zhang S. Wang S. Yin W. Liang M. Li J. Zhang Q.. (2014). Epidemic characteristics of hemorrhagic fever with renal syndrome in China, 2006-2012. BMC Infect. Dis. 14:384. 10.1186/1471-2334-14-384 25012160 PMC4105051

[B41] Zhang Y. Z. Zou Y. Fu Z. F. Plyusnin A. (2010). Hantavirus infections in humans and animals, China. Emerg. Infect. Dis. 16, 1195–1203. 10.3201/eid1608.090470 20678311 PMC3298307

[B42] Zuo S. Q. Fang L. Q. Zhan L. Zhang P. H. Jiang J. F. Wang L. P.. (2011). Geo-spatial hotspots of hemorrhagic fever with renal syndrome and genetic characterization of seoul variants in Beijing, China. PLoS Negl. Trop. Dis. 5:e945. 10.1371/journal.pntd.0000945 21264354 PMC3019113

